# Manually segmented vascular networks from images of retina with proliferative diabetic and hypertensive retinopathy

**DOI:** 10.1016/j.dib.2018.03.041

**Published:** 2018-03-15

**Authors:** Natasa Popovic, Miroslav Radunovic, Jelena Badnjar, Tomo Popovic

**Affiliations:** a Faculty of Medicine, University of Montenegro, Podgorica, Montenegro; b Faculty for Information Systems and Technologies, University of Donja Gorica, Podgorica, Montenegro

## Abstract

In this article we present a data set that contains 37 image files obtained by manual vessel segmentation of raw retinal images from Structured Analysis of the Retina (STARE) database (“The STARE Project”, 2018) [1]. Our expert segmented 8 images that are associated with the single diagnosis of hypertensive retinopathy and 9 images with the single diagnosis of proliferative diabetic retinopathy (Popovic et al., 2018) [2]. To validate the manual segmentation, the same expert additionally segmented a gold standard set of 20 raw images from the STARE database.

Raw images of retinas associated with either diabetic proliferative retinopathy or hypertensive retinopathy display the intricate and very different morphologies of retinal microvascular networks. Very frequently, they also have pathological changes such as exudates and hemorrhages. The presence of these changes, as well as neovascularization in proliferative diabetic retinopathy, poses a significant challenge for researchers who are developing automatic methods for retinal vessel segmentation. Therefore, this data set can be useful for the development of methods for automatic segmentation. In addition, the data can be used for development of methods for quantitation of microvascular morphology of the retina in various pathological conditions.

**Specifications table**

**Table:** 

Subject area	Physiology
More specific subject area	Microvascular remodeling
Type of data	Figures
How data was acquired	Manual vessel segmentation of raw color images of retina using Vampire software (“Vampire”, 2018) [3]
Data format	Portable Network Graphics (.png) black and white images, 700×605 pixel resolution
Experimental factors	Figures represent microvascular networks of 37 images from STARE database that were manually segmented by Natasa Popovic (NP).
Experimental features	• Seventeen images include all images from the STARE database that are associated with a single diagnosis of either hypertensive retinopathy or proliferative diabetic retinopathy that have acceptable image quality. • Additional 20 images were used for the validation of the manual segmentation by NP
Data source location	N/A
Data accessibility	Data is with this article

**Value of the data**

• This data set of 37 segmented images can be used by other researchers as a benchmark to validate newly developed computerized automatic methods of vessel segmentation.
• The high proportion of segmented images from retinas with hypertensive retinopathy (HR) and proliferative diabetic retinopathy (PDR) in this data set is an advantage because the presence of exudates, hemorrhages, neovascularization, and changes from laser treatment in the raw fundus images represents a significant challenge during the development of automatic methods for vessel segmentation, Fig. 1.

**Fig. 1:**
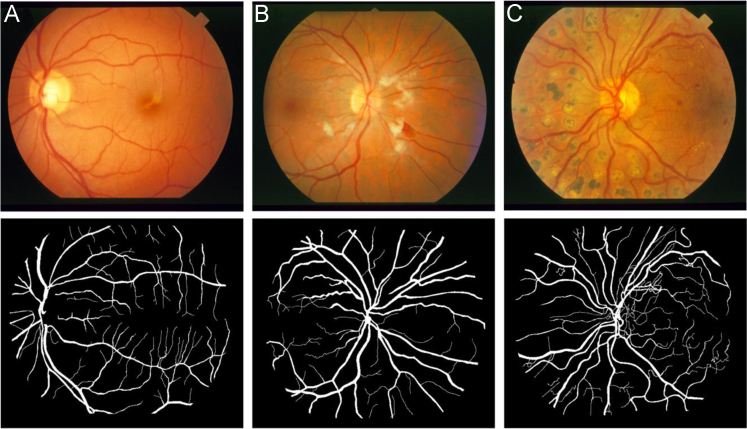
Vascular networks manually segmented by NP- bottom of each panel: A) normal retina, B) hypertensive retinopathy with exudates and hemorrhages, and C) proliferative diabetic retinopathy with changes from the laser treatment, hemorrhages, and neovascularization. Images at the top of each panel are representative raw images from the STARE database.
• The high proportion of segmented images from retinas with HR and PDR in this data set can be also used for the development of objective quantification methods that are able to detect differences in patterns of microvascular networks depending on ethiological factors that caused the microvascular remodeling.

## 1 Data

In this article we present a dataset that contains 37 image files obtained by manual vessel segmentation of raw retinal images from STARE database. Out of that, 8 images are associated with the diagnosis of HR, and 9 images with the diagnosis of PDR.

## 2 Experimental design, materials and methods

The STARE database is publicly available (“The STARE Project”, 2018) [1]. The complete database contains 402 raw color retinal images captured with a TopCon TRV-50 fundus camera at 35 degrees field of view. The diagnoses associated with each image are provided as well.

Examination of the complete STARE database revealed that 11 images are associated exclusively with the diagnosis of HR and no other diagnosis, while 21 images are associated only with the diagnosis of PDR. Next, images that had more than 25% of visual field obscured by glare, shadow, or gross artifact, out of focus images, as well as images captured far from the optic disc were excluded from the further analysis because of the poor quality.

**Fig. 2:**
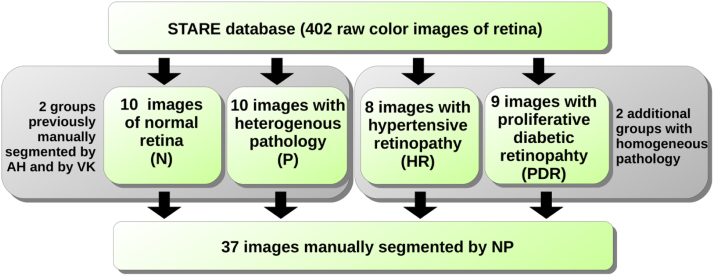
Dataset structure: 37 images manually segmented by NP are grouped by associated diagnosis.

Finally, our expert segmented 8 images that are associated with the single diagnosis of HR and 9 with the single diagnosis of PDR. To validate the manual segmentation, the same expert additionally segmented the gold standard set of 20 raw images from the STARE database that includes 10 images of normal retina and 10 images of retina associated with various pathologies, Fig. 2. This gold standard set was previously manually segmented by 2 other experts, Adam Hoover (AH) and Valentina Kouzentsova (VK), in the year 2000, and has been used as a benchmark for evaluating new methods for retinal vessel segmentation in numerous studies (Hoover et al., 2000) [4].

We performed the manual segmentation using Vampire software (“Vampire”, 2018) [3]. Following that, the validation of manual segmentation was done by visual inspection, by calculation of F1 score, and by calculation of fractal dimension as it was described in more detail previously (Popovic et al., 2018) [2].
