# Supplementary material for: Manually segmented vascular networks from images of retina with proliferative diabetic and hypertensive retinopathy
Source: Data Brief. 2018 Mar 15;18:470–3. doi: 10.1016/j.dib.2018.03.041 (PMC5996258; doi:10.1016/j.dib.2018.03.041)
Supplement: Supplementary file 1 — Supplementary material [file mmc1.pdf]

Manuscript DIB-D-18-00421

CONFLICTS OF INTEREST STATEMENT

The authors declare that they have no conflicting interests.

The corresponding author signs this declaration on behalf of all the authors

Natasa Popovic, MD, PhD

Natasa Popović MD, PhD
