# Supplementary figures and images for: Manually segmented vascular networks from images of retina with proliferative diabetic and hypertensive retinopathy
Source: Data Brief. 2018 Mar 15;18:470–3. doi: 10.1016/j.dib.2018.03.041 (PMC5996258; doi:10.1016/j.dib.2018.03.041)

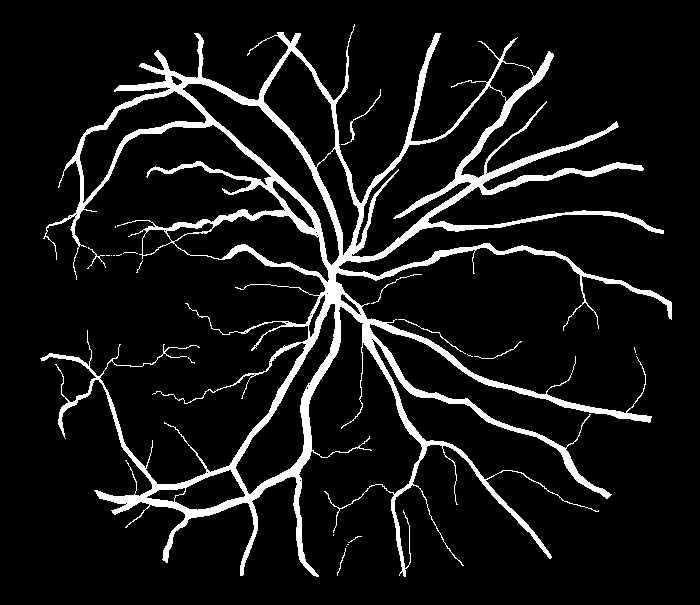

Supplement: Supplementary file 2 — Supplementary material [file mmc2.zip › data/HR048.png]

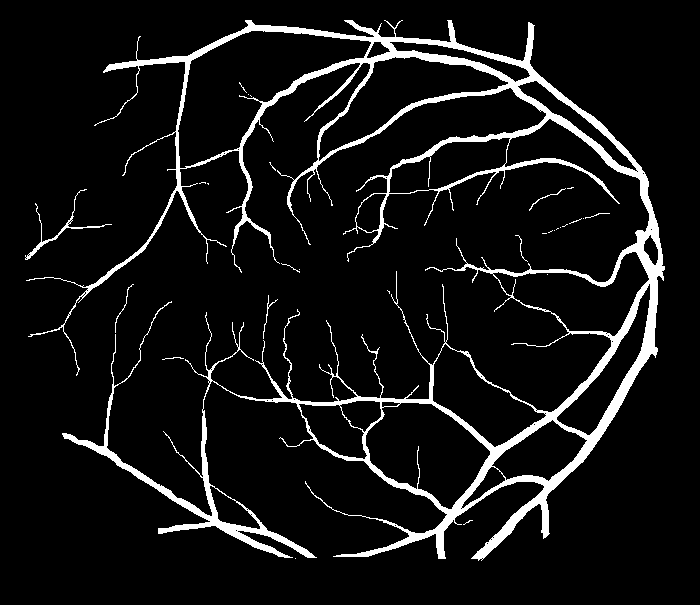

Supplement: Supplementary file 2 — Supplementary material [file mmc2.zip › data/HR075.png]

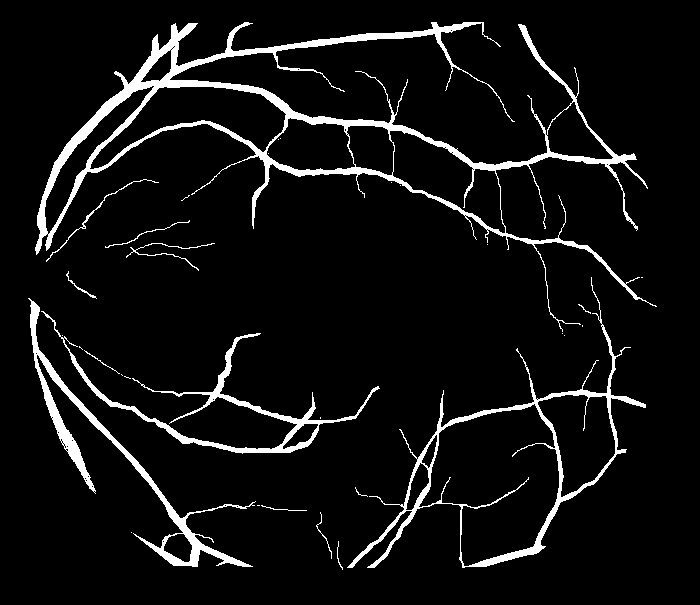

Supplement: Supplementary file 2 — Supplementary material [file mmc2.zip › data/HR090.png]

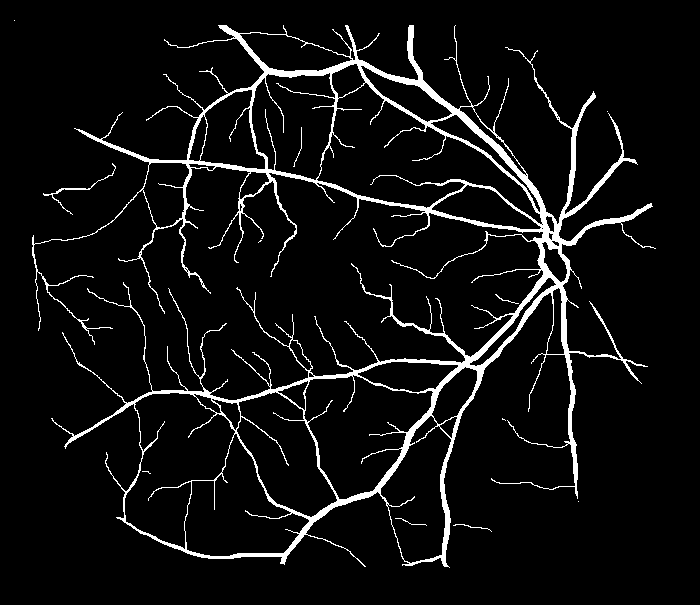

Supplement: Supplementary file 2 — Supplementary material [file mmc2.zip › data/HR220.png]

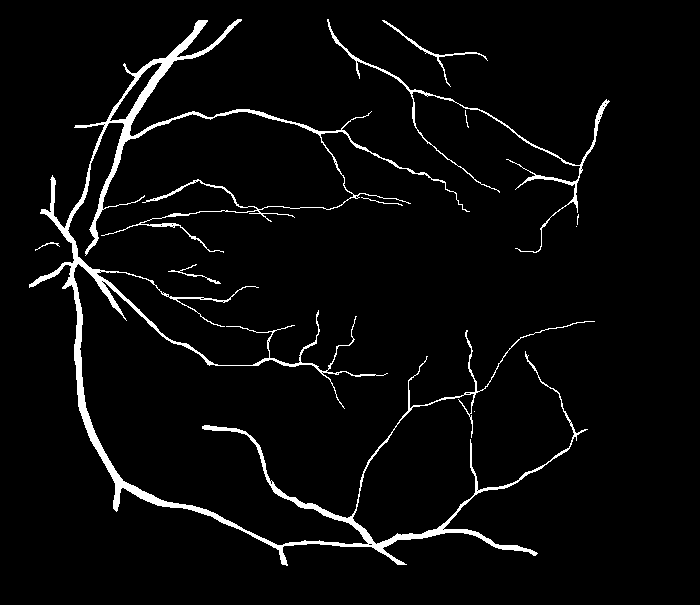

Supplement: Supplementary file 2 — Supplementary material [file mmc2.zip › data/HR271.png]

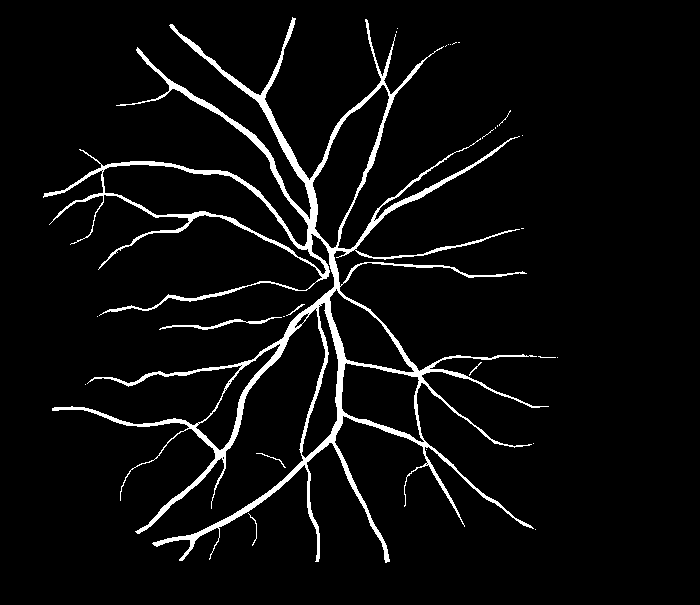

Supplement: Supplementary file 2 — Supplementary material [file mmc2.zip › data/HR272.png]

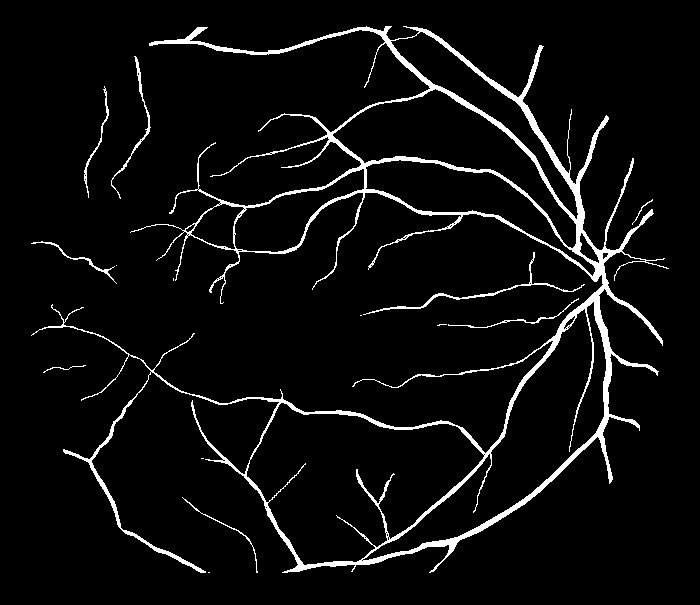

Supplement: Supplementary file 2 — Supplementary material [file mmc2.zip › data/HR275.png]

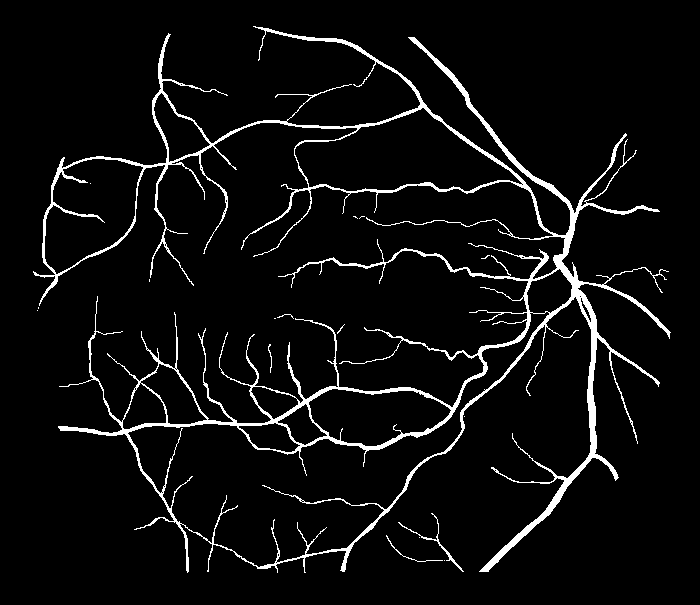

Supplement: Supplementary file 2 — Supplementary material [file mmc2.zip › data/HR397.png]

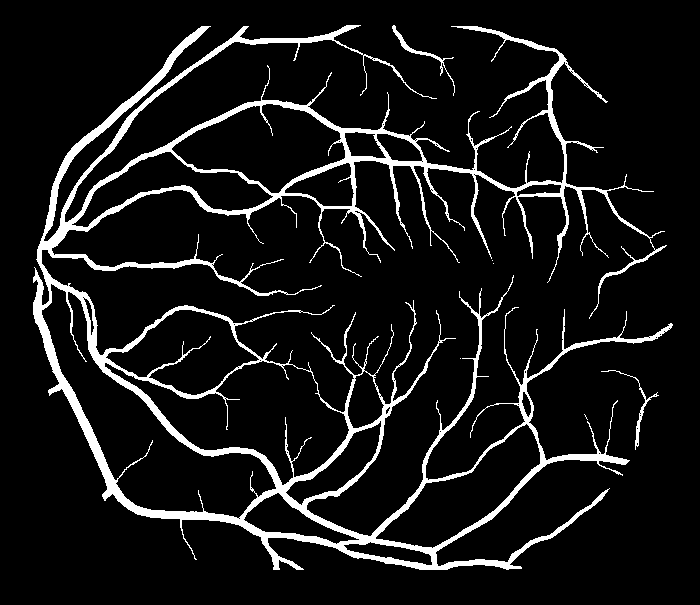

Supplement: Supplementary file 2 — Supplementary material [file mmc2.zip › data/N077.png]

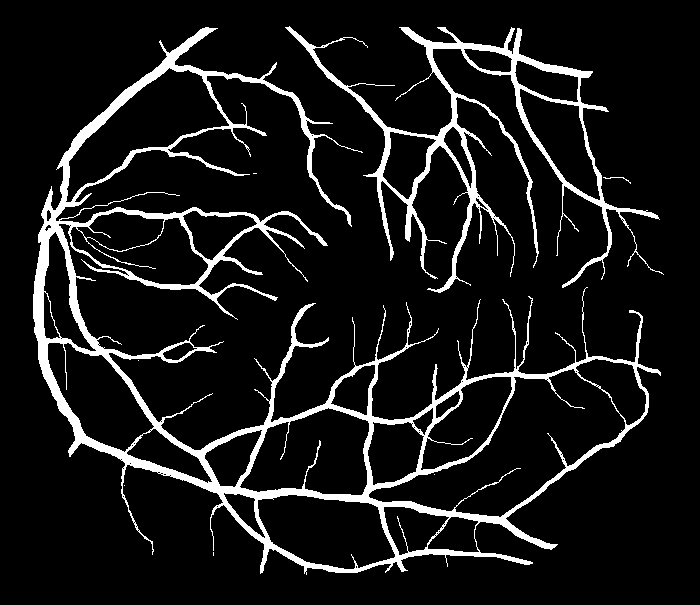

Supplement: Supplementary file 2 — Supplementary material [file mmc2.zip › data/N081.png]

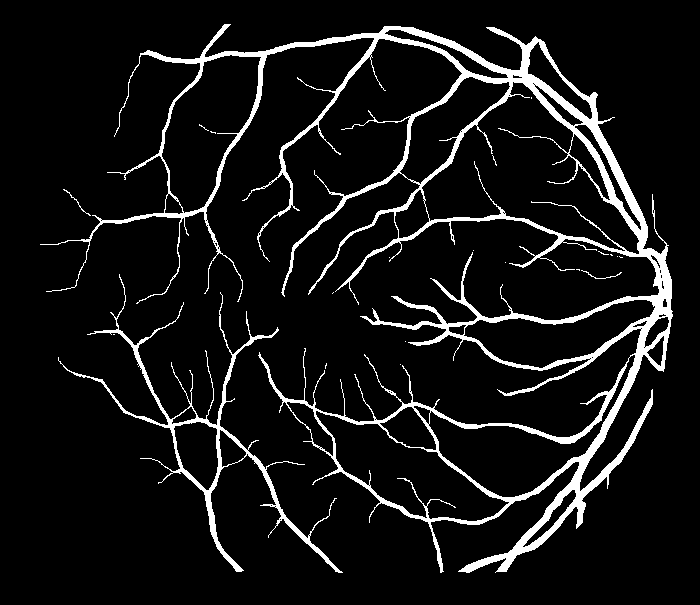

Supplement: Supplementary file 2 — Supplementary material [file mmc2.zip › data/N082.png]

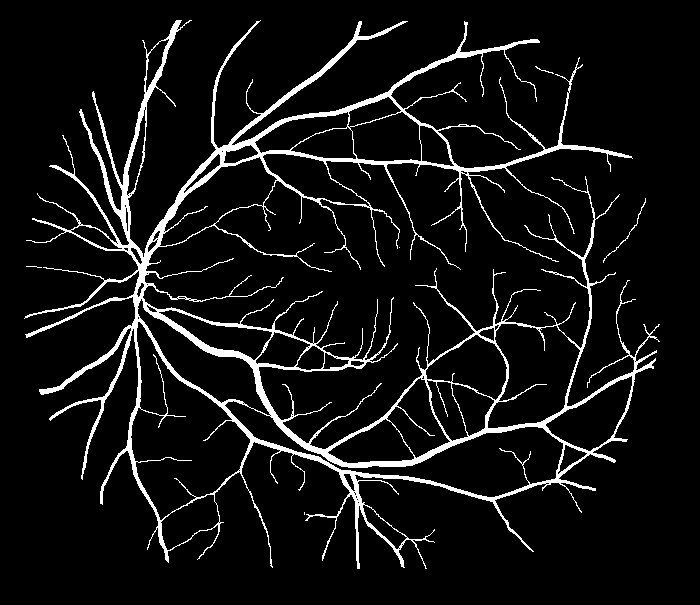

Supplement: Supplementary file 2 — Supplementary material [file mmc2.zip › data/N162.png]

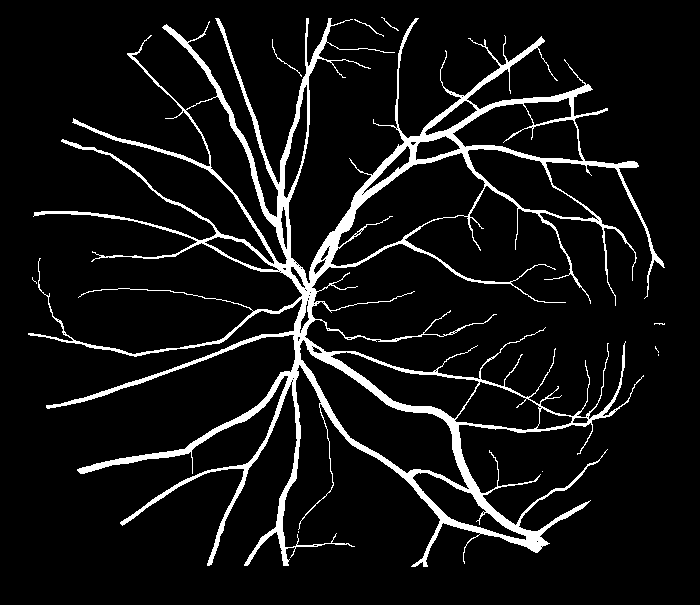

Supplement: Supplementary file 2 — Supplementary material [file mmc2.zip › data/N163.png]

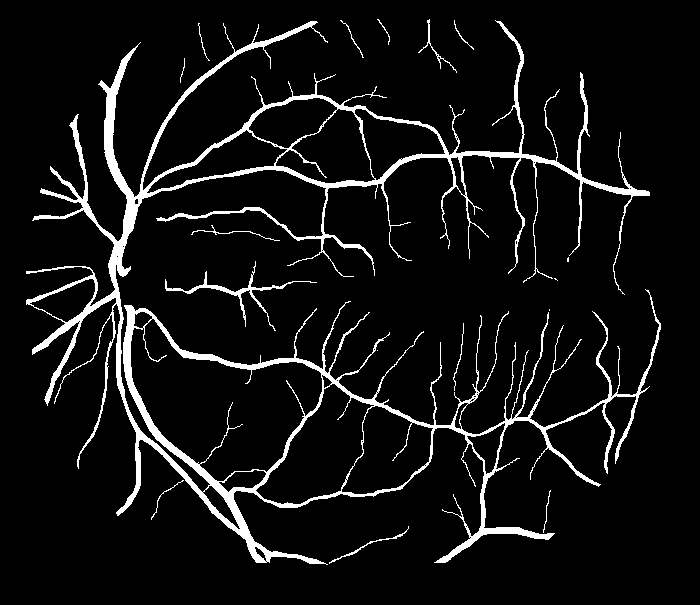

Supplement: Supplementary file 2 — Supplementary material [file mmc2.zip › data/N235.png]

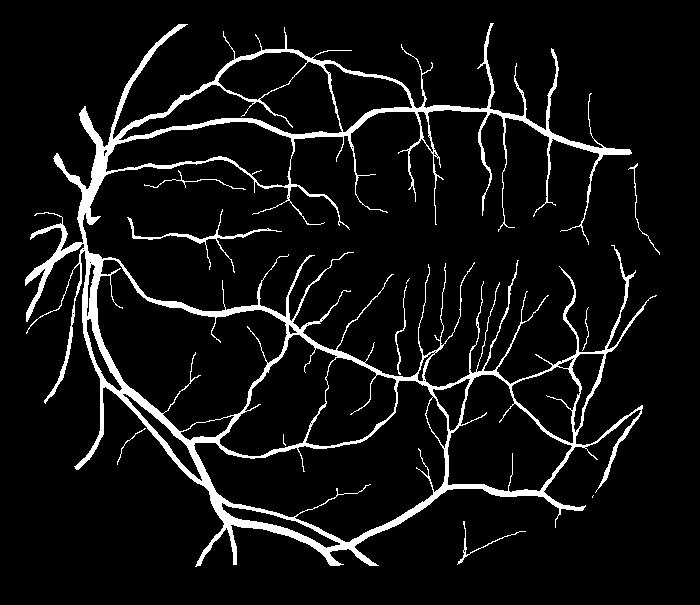

Supplement: Supplementary file 2 — Supplementary material [file mmc2.zip › data/N236.png]

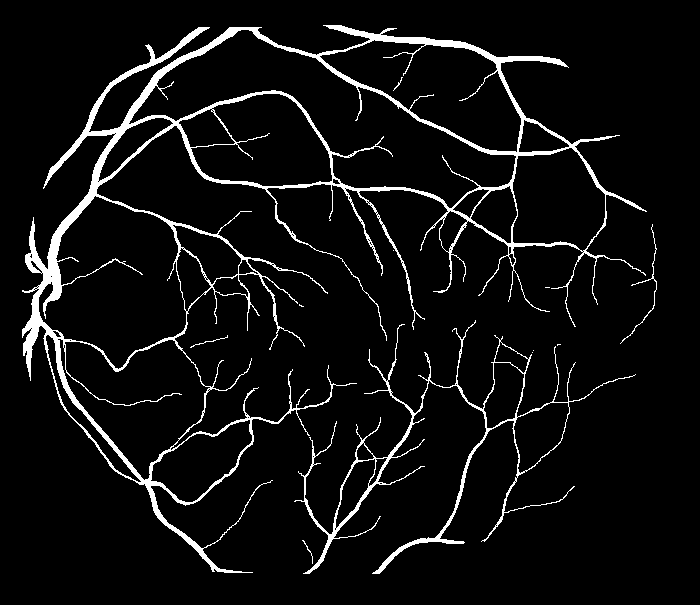

Supplement: Supplementary file 2 — Supplementary material [file mmc2.zip › data/N239.png]

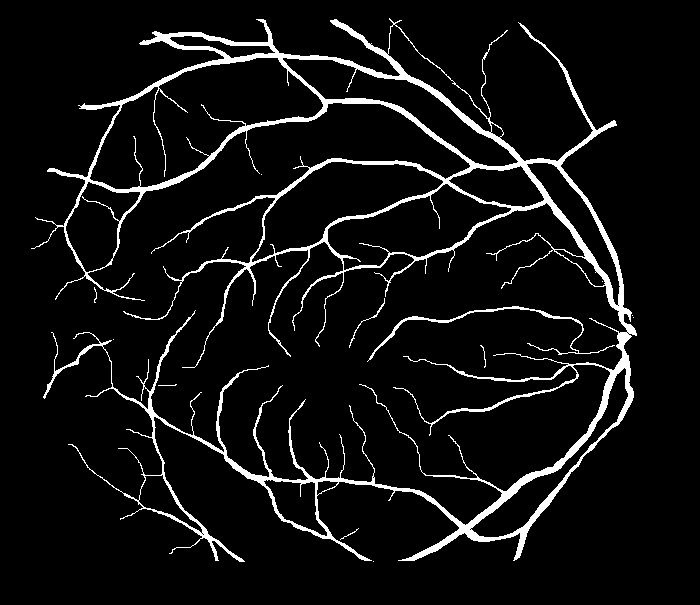

Supplement: Supplementary file 2 — Supplementary material [file mmc2.zip › data/N240.png]

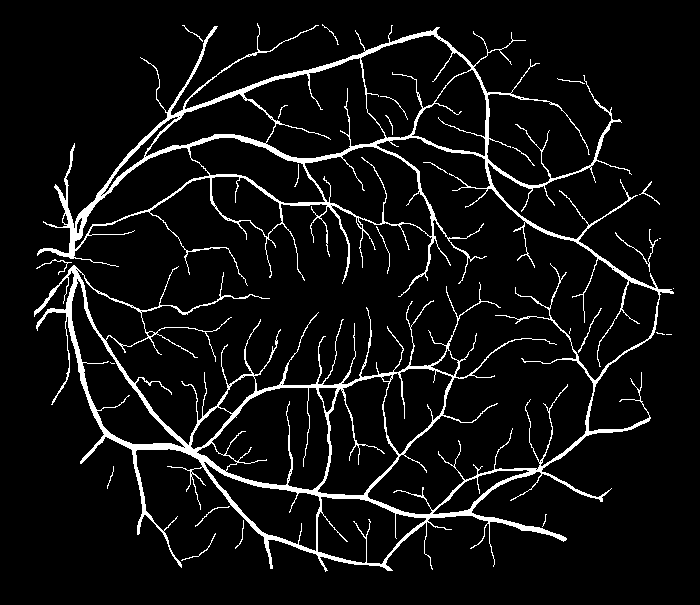

Supplement: Supplementary file 2 — Supplementary material [file mmc2.zip › data/N255.png]

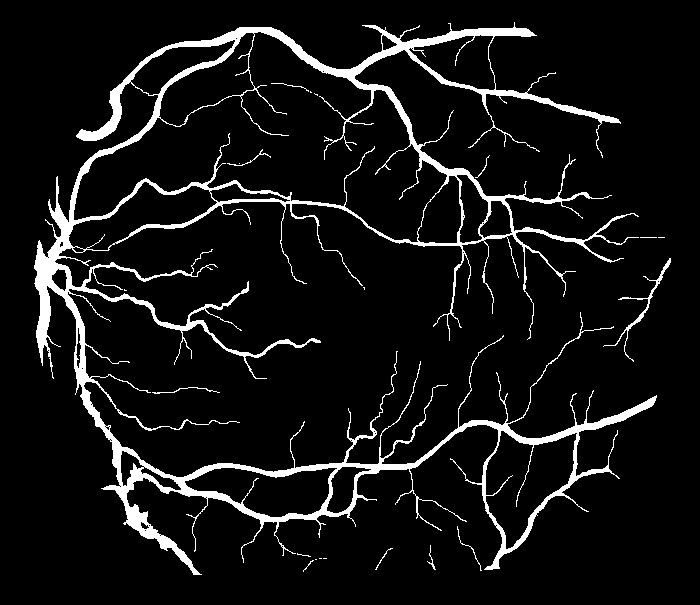

Supplement: Supplementary file 2 — Supplementary material [file mmc2.zip › data/P001.png]

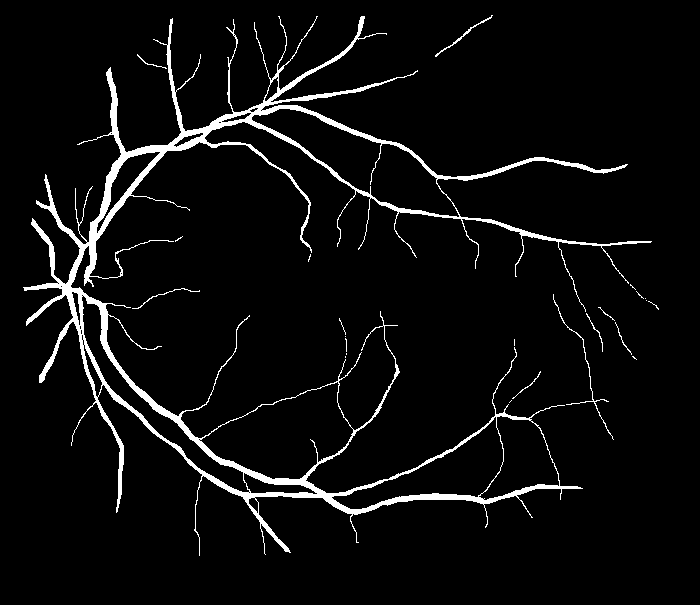

Supplement: Supplementary file 2 — Supplementary material [file mmc2.zip › data/P002.png]

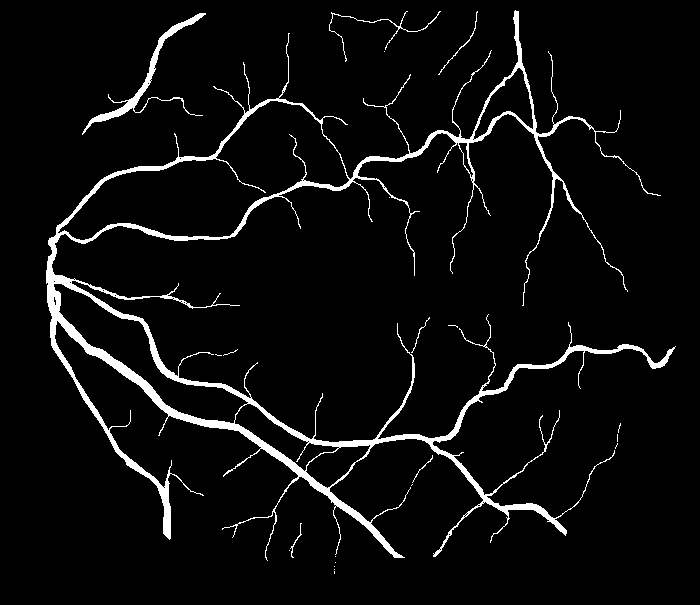

Supplement: Supplementary file 2 — Supplementary material [file mmc2.zip › data/P003.png]

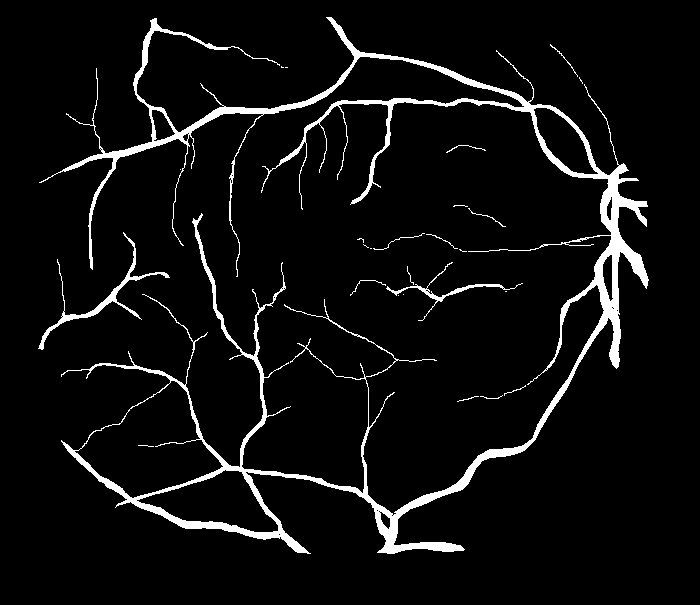

Supplement: Supplementary file 2 — Supplementary material [file mmc2.zip › data/P004.png]

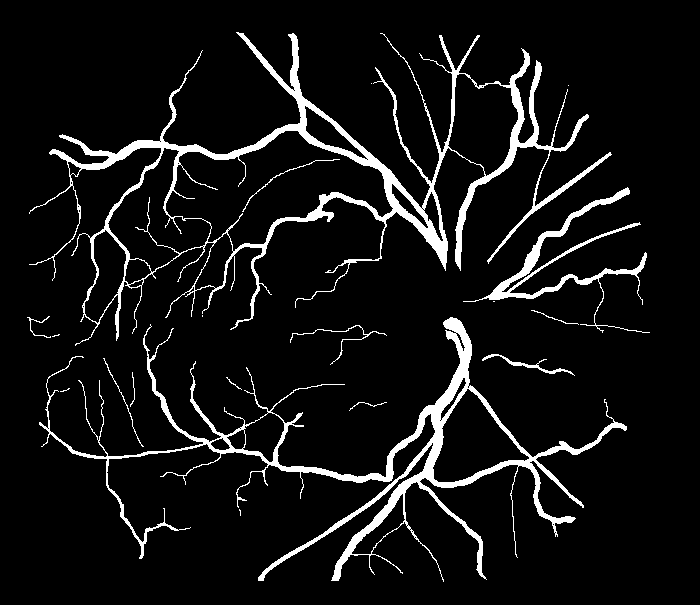

Supplement: Supplementary file 2 — Supplementary material [file mmc2.zip › data/P005.png]

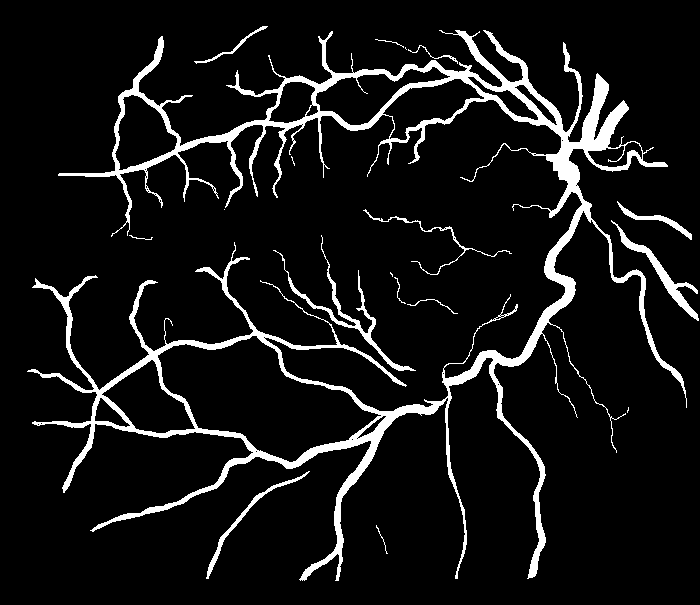

Supplement: Supplementary file 2 — Supplementary material [file mmc2.zip › data/P044.png]

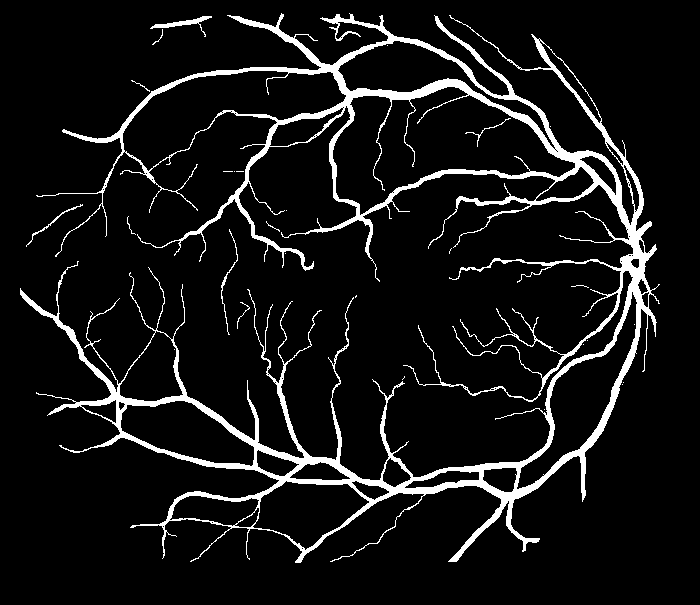

Supplement: Supplementary file 2 — Supplementary material [file mmc2.zip › data/P139.png]

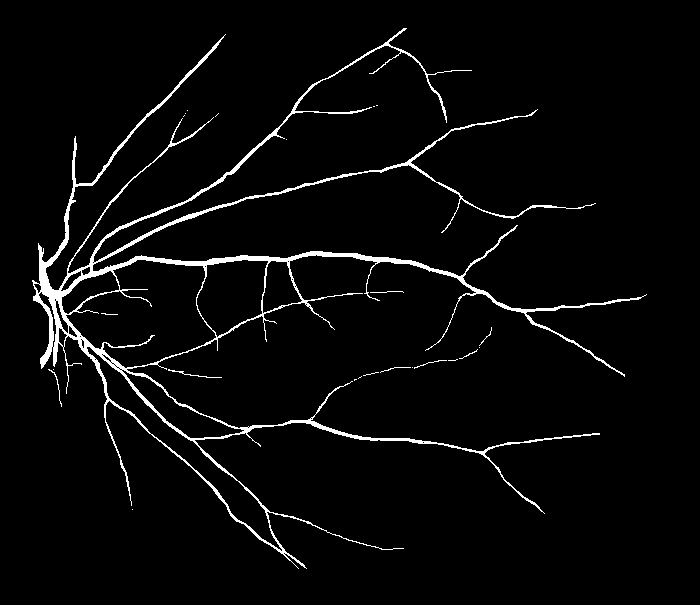

Supplement: Supplementary file 2 — Supplementary material [file mmc2.zip › data/P291.png]

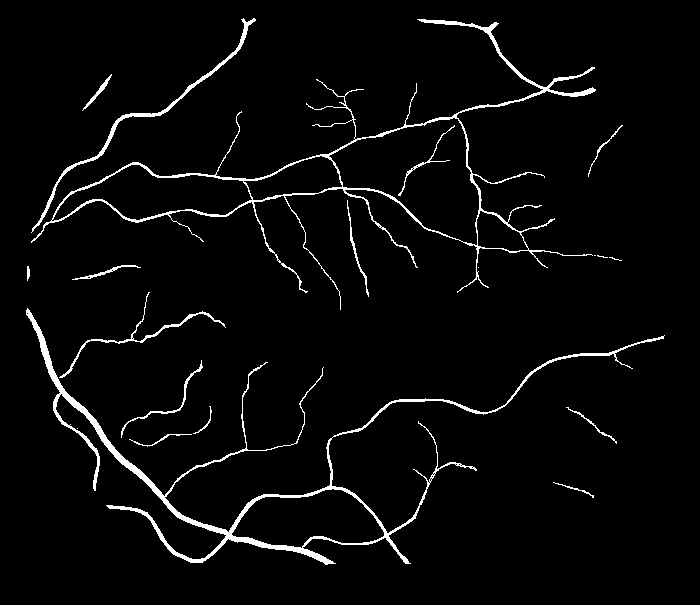

Supplement: Supplementary file 2 — Supplementary material [file mmc2.zip › data/P319.png]

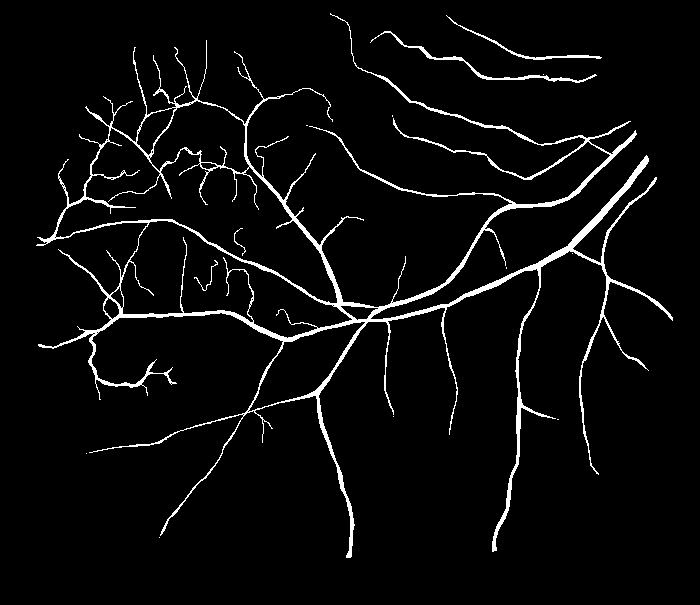

Supplement: Supplementary file 2 — Supplementary material [file mmc2.zip › data/P324.png]

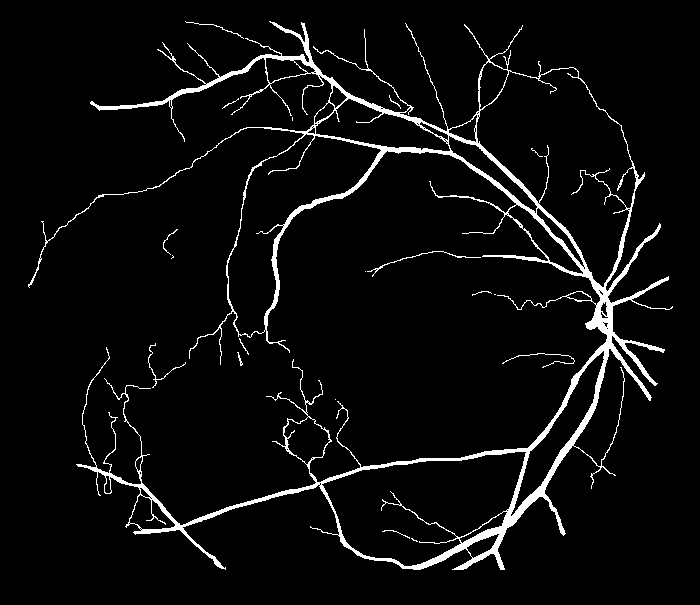

Supplement: Supplementary file 2 — Supplementary material [file mmc2.zip › data/PDR085.png]

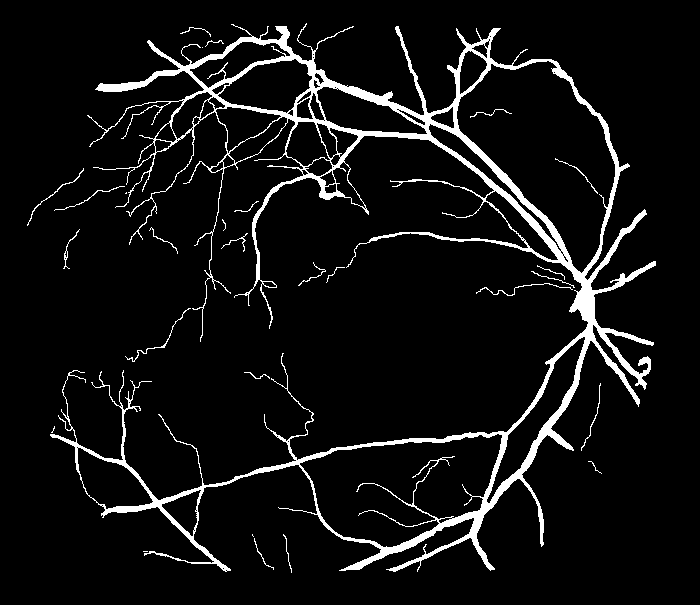

Supplement: Supplementary file 2 — Supplementary material [file mmc2.zip › data/PDR087.png]

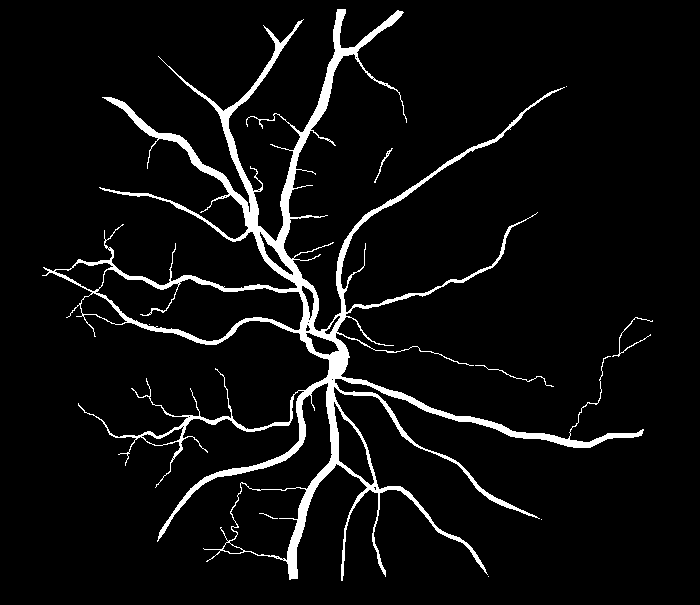

Supplement: Supplementary file 2 — Supplementary material [file mmc2.zip › data/PDR179.png]

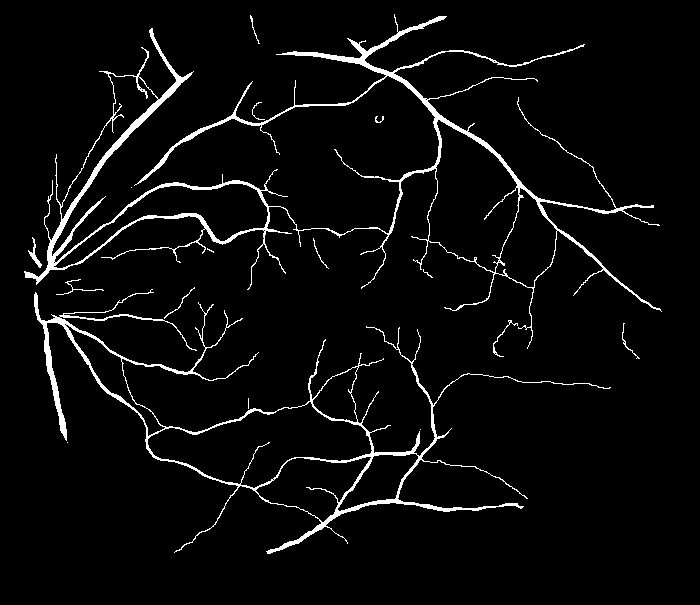

Supplement: Supplementary file 2 — Supplementary material [file mmc2.zip › data/PDR204.png]

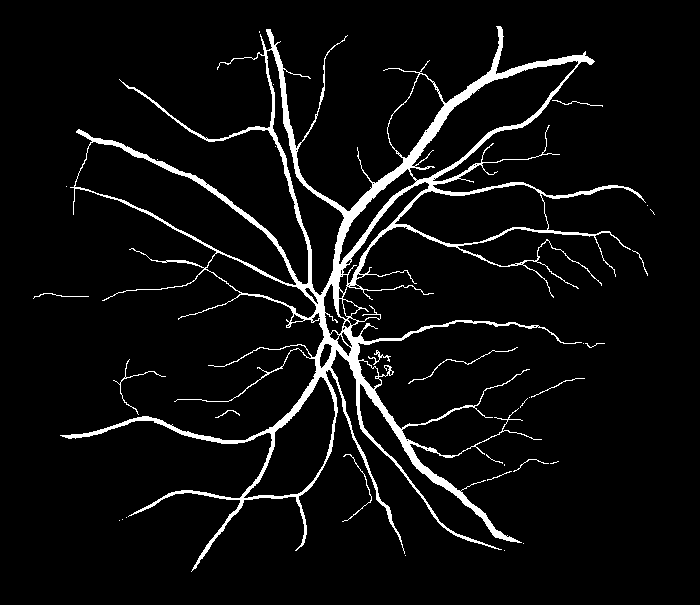

Supplement: Supplementary file 2 — Supplementary material [file mmc2.zip › data/PDR232.png]

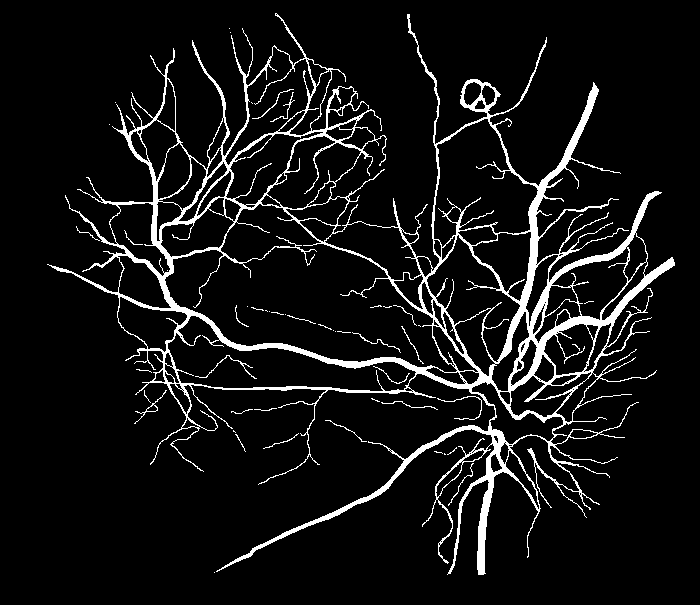

Supplement: Supplementary file 2 — Supplementary material [file mmc2.zip › data/PDR342.png]

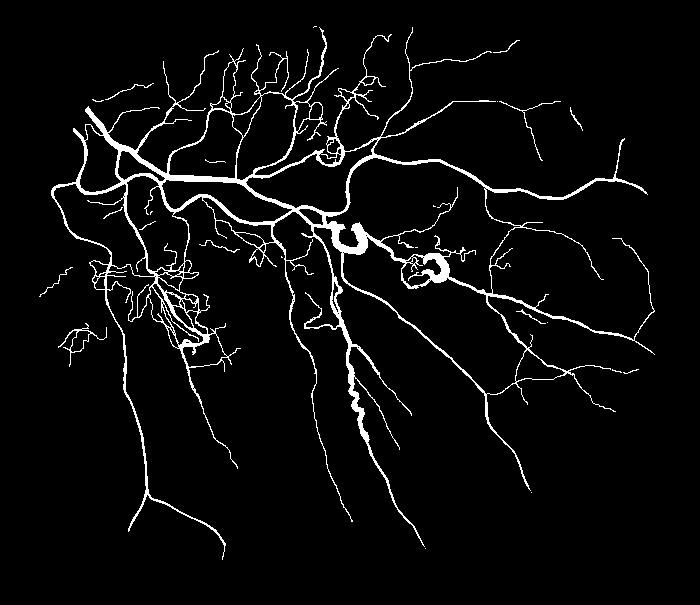

Supplement: Supplementary file 2 — Supplementary material [file mmc2.zip › data/PDR343.png]

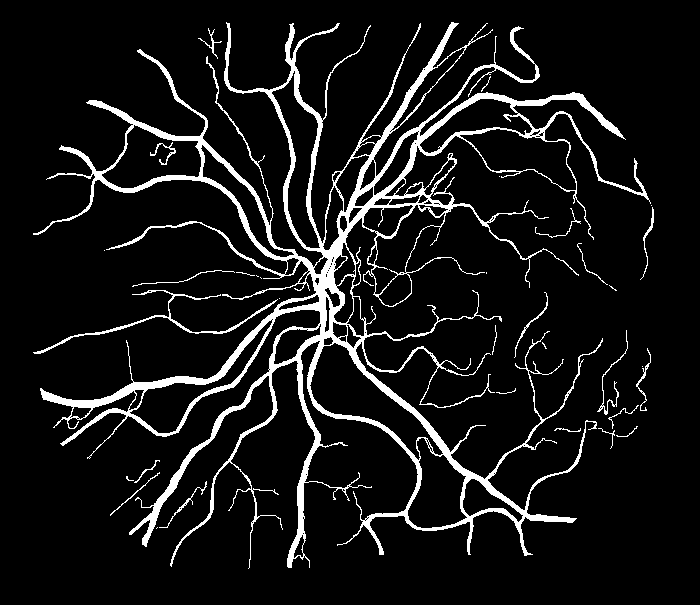

Supplement: Supplementary file 2 — Supplementary material [file mmc2.zip › data/PDR347.png]

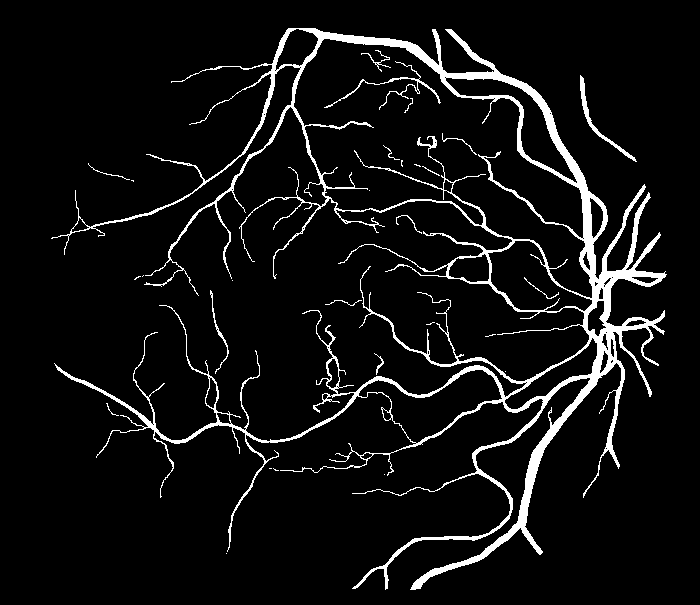

Supplement: Supplementary file 2 — Supplementary material [file mmc2.zip › data/PDR351.png]
